# Psychological distress in hypertension: Prevalence and links to self-care in Gonabad, Iran

**DOI:** 10.1371/journal.pone.0352892

**Published:** 2026-07-10

**Authors:** Fatemehzahra Naddafi, Alireza Jafari, Mahbobeh Nejatian, Hadi Tehrani

**Affiliations:** 1 Department of Geriatric Health, Faculty of Health, Tabriz University of Medical Sciences, Tabriz, Iran; 2 Student Research Committee, Tabriz University of Medical Sciences, Tabriz, Iran; 3 Department of Health Education and Health Promotion, School of Health, Nursing Research Center, Gonabad University of Medical Sciences, Gonabad, Iran; 4 Social Development and Health Promotion Research Center, Gonabad University of Medical Sciences, Gonabad, Iran; 5 Social Determinants of Health Research Center, Mashhad University of Medical Sciences, Mashhad, Iran; 6 Department of Health Education and Health Promotion, School of Health, Mashhad University of Medical Sciences, Mashhad, Iran; King Abdulaziz University Faculty of Medicine, SAUDI ARABIA

## Abstract

**Introduction:**

A critical component in the hypertension management and the prevention of its life-threatening complications, is self-care. Psychological factors such as stress, anxiety and depression may significantly influence self-care behaviors in people with hypertension. Therefore, this study aimed to investigate the prevalence of anxiety, depression and stress and their association with self-care levels among hypertensive patients.

**Methods:**

This analytical cross-sectional study was conducted in 2024−2025 in Gonabad, Iran, among a population of 509 hypertensive patients. Participants were selected through cluster random sampling, with inclusion criteria consisting of: A confirmed diagnosis of hypertension by a physician, Willingness to provide informed consent, Absence of cognitive impairment, and At least one year of residency in Gonabad. Data were gathered by three self-administered questionnaires: a demographic questionnaire, the DASS-21 (Depression, Anxiety, and Stress Scale), and the Self-Care of Hypertension Inventory (SC-HI V_3_). The collected data were analyzed using SPSS v25, with statistical tests including independent t-tests, ANOVA, Multiple Linear Regression and Pearson’s correlation.

**Results:**

The majority of participants in this study were female and older persons (age > 60 years). Marital status showed a significant association with levels of depression, anxiety, and stress. Additionally, variables such as age group, marital status, education level, and occupation were significantly correlated with hypertension self-care levels and all three of its subscales. Furthermore, 62.5%, 75.2%, and 59.3% of hypertensive patients exhibited depression, anxiety, and stress, respectively. Pearson’s correlation analysis revealed a significant negative association between: Depression and self-care (r = −0.314, p < 0.001), Anxiety and self-care (r = −0.330, p < 0.001). A significant negative correlation was also observed between depression/anxiety and the age at hypertension onset. Variables of Depression, Anxiety, Stress, Age, Education and Income could predict 15% of self-care of hypertension variance.

**Conclusion:**

The prevalence of psychological disorders—particularly stress, depression, and anxiety—among hypertensive patients is alarmingly high. Moreover, significant negative correlations exist between these mental health conditions and hypertension self-care levels. These findings underscore the critical need for: Preventive interventions and Mental health promotion programs tailored to this population. Policymakers, healthcare providers, and researchers must prioritize integrating psychological support into hypertension management strategies to mitigate these adverse effects and improve patient outcomes.

## Introduction

Hypertension affects approximately 1.28 billion people worldwide and 25% of Iranians – equating to 1 in 4 adults [1–3]. Hypertension described as sustained diastolic pressure ≥90 mmHg or systolic blood pressure ≥140 mmHg [4,5], this “silent killer” represents a major risk factor for cardiovascular disease, stroke, and renal disorders [6,7]. Uncontrolled hypertension may lead to severe complications including cerebrovascular accidents, myocardial infarction, renal failure, vascular rupture, vision loss, and cognitive impairment [8].

Poor adherence to self-care practices constitutes the primary barrier to effective hypertension control [8]. Self-care is the first and most effective step to control and improve high blood pressure, and successful management of hypertension requires continued self-care behaviors [9,10]. As the cornerstone of hypertension management, self-care encompasses voluntary, adaptive behaviors to restore, maintain, or enhance health [11]. For hypertensive patients, this includes: Medication adherence, Blood pressure monitoring, Low-sodium/low-fat dietary compliance, Smoking cessation, Alcohol moderation, Weight management, Regular physical activity, Stress reduction, Routine health screenings [8,12]. Inadequate self-care correlates with elevated risk of cardiovascular and cerebrovascular events and increased hospitalization rates [13], underscoring the imperative for enhanced self-care to improve blood pressure control and reduce complications [14].

Psychological distress significantly undermines self-care behaviors, contributing to poor long-term hypertension control and reduced treatment adherence [15]. Depression, stress, and anxiety are most prevalent in chronic diseases such as hypertension [16]. Patients with hypertension demonstrate significant vulnerability to anxiety and depression. Abdisa et al.’s Ethiopian study revealed that over 25% of hypertensive patients reported clinically significant depressive and anxiety symptoms [17]. Similarly, Kretchy et al.’s Ghanaian research found notable prevalence rates of 56% for anxiety, 20% for stress, and 4% for depression among this population [18]. Also results of a study by Ranjbar kouchaksaraei et al. in Iran among hypertensive patients indicated that 54% and 38% of them have type A and type B personality, respectively [19]. Also according to a 10-year longitudinal study, there was relationship between hostility and diastolic blood pressure [20]. Results of another study suggested that there is significant difference between hypertensive and healthy people in psychosis and neurosis traits and Emotion-oriented coping style. Also hypertensive people with essential hypertension are anxious, worried, aggressive and susceptible to depression [21].

These mental health comorbidities (depression, anxiety, stress) exert particularly detrimental effects on treatment adherence behaviors [18,22].Among the various factors influencing hypertension self-care, psychological disturbances appear to play a disproportionately prominent role [16]. This relationship was quantitatively demonstrated in Iran by Eghbali et al., who documented significantly higher self-care among patients with lower psychological distress levels [16].

The crucial importance of self-care in hypertension management, coupled with the high prevalence and substantial impact of depression, anxiety, and stress on self-care behaviors, underscores the necessity of this investigation. Our comprehensive literature review reveals two significant research gaps in the Iranian context: No prior study has systematically examined both the prevalence of these psychological factors and their interrelationships with self-care in hypertensive patients and Existing research, including Eghbali et al.’s study [16], has not employed condition-specific instruments for assessing hypertension self-care and psychological distress. To address these limitations, this study was carried out in 2024 to: Determine the prevalence of depression, anxiety, and stress and Evaluate their association with self-care levels among hypertensive patients in Gonabad, Iran, using validated, disease-specific measurement tools.

## Methods

This cross-sectional analytical study was performed to assess the prevalence of stress, depression and anxiety and their association with self-care levels among hypertensive patients in Northeast Iran (Gonabad) in 2025.

### Sampling method

Participants were selected through population-proportionate cluster sampling in Gonabad (February 2, 2025 to May 9 2025). The sampling process involved: Identification of all comprehensive health service centers, Determination of the hypertensive people number at each center, Treatment of each center as a cluster, with sample size allocation proportional to cluster population and Random selection of eligible participants meeting inclusion criteria from each cluster. Trained researchers visited the health centers to: Distribute questionnaires in person, collect self-reported data from literate participants and Assist illiterate participants through interviewer-administered questionnaires Inclusion Criteria include: Physician-confirmed hypertension diagnosis, Willingness to provide written informed consent, Absence of cognitive impairment and Minimum one-year residency in Gonabad. Exclusion Criteria include: Incomplete questionnaires (participants with excessive missing data were excluded during analysis).

### Sample size calculation

Due to the absence of prior studies directly measuring hypertension self-care prevalence in Iran, we calculated the maximum required sample size using: Prevalence proportion (p) = 0.5, 95% confidence level, 80% statistical power, 0.066 precision and 10% nonresponse rate. The final calculated sample size was 495 participants, settled using the following formula:


$$\begin{array}{c} \frac{{\left(z_{\text{1}-\frac{\alpha }{\text{2}}}\;+z_{\text{1}-\beta }\right)}^{\text{2}}\;p(\text{1}-p)\;}{{\left(d\right)}^{\text{2}}}=\frac{{\left(\text{2}.\text{8}\right)}^{\text{2}}\;\text{0}.\text{5}(\text{0}.\text{5})\;}{{\left(\text{0}.\text{066}\right)}^{\text{2}}}=\text{450}\times \text{10 }\%\;nonresponse\;rate=\text{495} \end{array}$$

### Data collection tools

The data collection tools in this study comprised a demographic variables questionnaire, the DASS-21, and the SC-HI V3.

### Demographic variables questionnaire

This section examined variables such as marital status, education level, gender, age, occupational status, hypertension treatment modalities, and participants’ comorbidities.

### Depression, Anxiety, and Stress Scale-21 (DASS-21)

This study employed the DASS-21, consisting of three subscales (7 items per subscale) and 21 items. This tool assesses the prevalence of depressive, anxious, and stress-related symptoms over the preceding weeks. Data were obtained using a Likert-type scale with four response options, ranging from 0 (“Not at all”) to 3 (“Most of the time”). Subscale scores were calculated by summing item scores, with a maximum possible score of 21 per subscale. Higher scores indicate greater psychological distress. High reliability for the DASS-21 was established in the original validation study, with Cronbach’s alpha coefficients of 0.84 (anxiety), 0.90 (stress), and 0.91 (depression) [23,24]. In this study the Cronbach’s alpha of DASS-21 was 0.942.

### Self-care of hypertension inventory version_3_ (SC-HI V_3_)

The development of this questionnaire was carried out by Dickson et al. in 2021. SC-HI V3 has 23 items and 3 subscales including maintenance (9 items), monitoring (7 items), and management (7 items). Each item is rated on a 5-point Likert scale. The responses to this questionnaire are also rated on a 5-point Likert scale from never and rarely (1), sometimes (2,3,4), and daily and always (5) in the maintenance and monitoring subscales, as well as not unlikely (1), somewhat likely (2,3,4), and very likely (5) in the management subscale. This inventory includes 12 recommended behaviors in hypertension self-care and is based on the recommendations of the American Heart Association, studies, and clinical guidelines. A higher score indicates a higher level of self-care, and a lower score indicates a lower level of self-care for the patient [25,26]. The score of each subscale was calculated according to the tool developers’ instructions using the formula (Actual raw score-lowest possible raw score)/(possible raw score range) [27]. For this research, the overall score was calculated by aggregating the subscale scores. Previous validation in an Iranian sample reported a Cronbach’s alpha of 0.879 for the tool [28]. In this study the Cronbach’s alpha of SC-HI V3 was 0.904.

### Statistical analysis

Prior to analysis, the distribution of the data was evaluated for normality using kurtosis and skewness indices. Given the normality of the data, parametric statistical tests (one way ANOVA, independent samples t-test and Pearson correlation) were used. Data analysis was conducted using SPSS version 25, adopting a significance level of p < 0.05. Also Multiple Linear Regression model was employed to determine self-care predicting factors and control for confounding variables such as age, income, and education.

### Ethics approval and consent to participate

This study is based on a research project approved by Ethics Committee of Mashhad University of Medical Sciences with the code of ethics IR.MUMS.REC.1403.363. All procedures performed in this study were in accordance with the ethical standards of the institutional and/or national research committee and with the 1964 Helsinki declaration and its later amendments or comparable. Written Informed Consent was obtained from all subjects

## Results

In this study, Participants had an average age of 60.40 years and a standard deviation of 13.09 Most of the participants were the age of ≥60 (n = 266), the woman (n = 348), married (n = 390), with the elementary education (n = 158), housewife (n = 286), resident in urban areas (n = 349), and with medium income (n = 354). The age onset of hypertension in most participants was between 30–60 (n = 327) and 43.4% (n = 204) reported that their duration period of hypertension was more than 10 years (Table 1).

**Table 1:** Description of demographic variables.

Variables		Data (n = 509)	
		n	%
**Sex**	Men	155	30.8
	Women	348	69.2
**Age group**	<30	5	1
	30-60	226	45.5
	≥60	266	53.5
**Marital status**	Married	390	78.2
	Single	7	1.4
	Divorced	9	1.8
	Widowhood	93	18.6
**Education level**	Illiterate	86	17.5
	Elementary	158	32.1
	Middle school	56	11.4
	High school	15	3.0
	Diploma	71	14.4
	Academic degree	106	21.5
**Occupation**	Housewife	286	57.5
	Employed	37	7.4
	Retired	121	24.3
	Self-employed	37	7.4
	labor	16	3.2
**Inhabitant**	Urban	349	68.6
	Rural	103	20.2
	Outskirts	14	2.8
**Income**	Good	13	2.8
	Medium	354	76.8
	Weak	94	20.4
**The age onset of hypertension**	<30	34	7.3
	30-60	327	70.0
	≥60	106	22.7
**Duration period of hypertension**	≤ 5	152	32.3
	6-10	114	24.3
	>10	204	43.4
**BMI**	<18.5	3	0.8
	18.5-24.99	103	26.1
	25-29.99	177	44.8
	≥30	112	28.4

**Fig 1:**
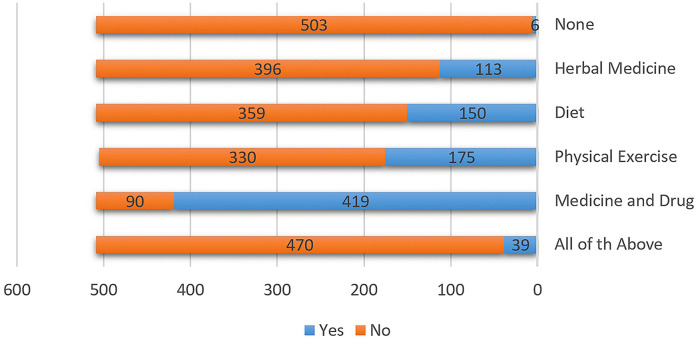
Treatment modalities for hypertension management.

Most people reported that the most treatment modalities that the used for hypertension management were used the medicine/ drug, physical exercise, and diet (Fig 1).

**Fig 2:**
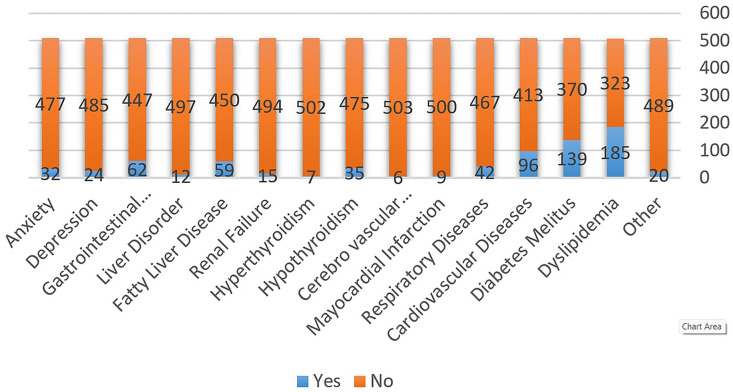
Comorbidities in hypertensive patients.

Among the various comorbidities reported, dyslipidemia was the most prevalent (n = 185), and cerebrovascular disease (n = 6) and hyperthyroidism (n = 7) were the least prevalent (Fig 2).

The associations between the states of depression, anxiety, stress and demographic variables are displayed in Table 2. Based on the results, marital status had a significant relationship with depression, anxiety, and stress (p < 0.001). A significant association was observed between the Inhabitant and depression (p = 0.002) and anxiety (p = 0.003). A significant association was observed between Income status and depression, anxiety, and stress (p < 0.001). A significant association was observed between the age at hypertension onset and anxiety (p = 0.026) and stress (p = 0.047). Duration period of hypertension had only significant relationship with anxiety (p = 0.012).

**Table 2:** Relationship between demographic variables and psychological status (depression, anxiety, stress).

Variables		DASS-21 *Mean (SD)*					
		Depression	P-value	Anxiety	P-value	Stress	P-value
Sex*	Men	15.29 (10.79)	0.214	15.08 (10.34)	0.570	17.20 (10.02)	0.651
	Women	14.02 (10.52)		14.56 (9.08)		16.75 (10.25)	
Age group**	<30	21.30 (10.07)	0.242	22.00 (8.60)	0.220	25.37 (7.78)	0.152
	30-60	14.70 (10.61)		14.54 (9.48)		16.92 (10.21)	
	≥60	13.93 (10.47)		14.74 (9.45)		16.57 (10.00)	
Marital status**	Married	13.39 (9.92)	< 0.001	13.72 (8.85)	< 0.001	16.10 (9.84)	< 0.001
	Single	26.64 (11.71)		27.69 (8.51)		29.14 (7.28)	
	Divorced	25.33 (6.70)		25.11 (6.64)		24.88 (6.17)	
	Widowhood	16.27 (12.02)		16.65 (10.61)		18.01 (11.08)	
Education level**	Illiterate	14.99 (9.94)	0.351	15.66 (8.86)	0.392	17.34 (8.93)	0.751
	Elementary	13.02 (10.82)		14.49 (9.59)		16.09 (11.01)	
	Middle school	15.39 (10.96)		15.38 (10.23)		16.89 (9.59)	
	High school	16.00 (9.79)		16.53 (9.75)		19.46 (7.61)	
	Diploma	13.83 (10.96)		12.73 (8.99)		17.73 (11.25)	
	Academic degree	15.67 (10.11)		15.24 (9.54)		17.20 (9.46)	
Occupation**	Housewife	13.86 (10.54)	0.424	14.57 (9.03)	0.913	16.39 (10.34)	0.368
	Employed	15.05 (10.30)		15.81 (10.32)		17.12 (9.47)	
	Retired	14.86 (10.47)		14.58 (10.02)		17.22 (10.20)	
	Self-employed	16.54 (11.55)		14.96 (10.64)		19.27 (10.06)	
	labor	11.40 (10.42)		13.24 (8.18)		13.74 (9.26)	
Inhabitant **	Rural	12.30 (10.31)	0.002	14.25 (8.47)	0.003	15.54 (10.07)	0.074
	Urban	14.55 (10.44)		14.44 (9.54)		17.03 (10.06)	
	Outskirts	22.31 (9.61)		23.04 (9.57)		21.77 (9.03)	
Income**	Good	16.80 (6.98)	< 0.001	14.48 (8.04)	< 0.001	20.02 (7.13)	< 0.001
	Medium	12.41 (9.97)		13.50 (8.88)		15.41 (9.81)	
	Weak	21.15 (10.38)		19.86 (10.51)		22.18 (10.02)	
The age of Hypertension onset **	<30	18.50 (10.63)	0.058	18.69 (9.31)	0.026	20.84 (9.14)	0.047
	30-60	13.95 (10.24)		14.06 (9.52)		16.31 (9.98)	
	≥60	14.05 (11.66)		14.76 (9.54)		16.56 (11.01)	
Duration period of Hypertension**	≤ 5	13.59 (11.13)	0.458	12.66 (9.41)	0.012	16.10 (10.86)	0.544
	6-10	13.94 (10.55)		14.90 (10.06)		16.39 (10.42)	
	>10	14.96 (10.40)		15.67 (9.22)		17.25 (9.71)	
BMI**	<18.5	12.66 (7.57)	0.321	16.63 (12.00)	0.451	26.80 (5.25)	0.369
	18.5-24.99	16.37 (11.73)		16.14 (10.60)		17.99 (10.31)	
	25-29.99	15.40 (10.52)		15.56 (9.64)		17.24 (10.02)	
	≥30	13.79 (9.39)		14.11 (8.82)		17.14 (9.37)	

* Independent Samples T-test, ** One-way ANOVA Test.

Table 3 showed the relationship between demographic variables and self-care of hypertension and its domains (Maintenance, Monitoring and Management). Based on the results, age group had a significant relationship with self-care (p < 0.001) and domains of maintenance (p < 0.001), monitoring (p = 0.002), and management (p = 0.001). The age at hypertension onset was significantly associated with overall self-care (p < 0.001) and its maintenance (p < 0.001), monitoring (p = 0.001), and management (p < 0.001) domains. Marital status had a significant relationship with self-care (p < 0.001) and subscales of maintenance (p < 0.001), monitoring (p = 0.030), and management (p = 0.002). A significant association was found between education level and self-care (p < 0.001), including its monitoring (p = 0.004), maintenance (p < 0.001) and management (p = 0.013) domains. Occupation had a significant relationship with self-care (p = 0.007) and subscales of maintenance (p = 0.021), monitoring (p = 0.017), and management (p = 0.019). The maintenance (p = 0.034) and management (p = 0.003) aspects of self-care, along with the overall score (p = 0.025), demonstrated significant relationships with the inhabitants’ residential area. BMI had a significant relationship only with domain of monitoring (p = 0.016).

**Table 3:** Relationship between demographic variables and self-care of hypertension and its subscales (maintenance, monitoring and management).

Variables		SC-HI V_3_ *Mean (SD)*							
		Maintenance	P-value	Monitoring	P-value	Management	P-value	Total Self-care	P-value
Sex*	Men	69.12 (18.89)	0.150	63.14 (20.48)	0.150	58.21 (21.25)	0.511	74.33 (14.63)	0.180
	Women	71.64 (16.02)		65.87 (17.53)		59.49 (19.48)		76.16 (12.71)	
Age group**	<30	47.85 (20.60)	< 0.001	50.71 (23.47)	0.002	32.14 (23.69)	0.001	57.60 (15.50)	<0.001
	30-60	68.66 (18.58)		62.63 (19.01)		57.08 (20.71)		73.71 (14.10)	
	>60	73.28 (14.92)		67.69 (17.20)		61.13 (19.06)		77.59 (12.18)	
Marital status**	Married	71.87 (16.64)	< 0.001	65.84 (18.08)	0.030	60.06 (18.71)	0.002	76.38 (12.63)	<0.001
	Single	59.37 (19.85)		53.06 (31.97)		43.36 (26.83)		64.62 (19.40)	
	Divorced	49.20 (24.20)		50.99 (26.34)		38.88 (32.65)		59.94 (21.27)	
	Widowhood	69.89 (16.70)		64.70 (17.32)		58.28 (22.50)		75.00 (14.07)	
Education level**	Illiterate	73.12 (12.97)	< 0.001	65.30 (17.56)	0.004	62.83 (18.00)	0.013	77.35 (11.25)	<0.001
	Elementary	73.25 (16.41)		68.05 (16.82)		61.09 (18.18)		77.67 (12.18)	
	Middle school	72.52 (17.59)		68.31 (20.35)		58.84 (22.39)		76.91 (14.92)	
	High school	59.03 (23.12)		58.99 (21.71)		46.22 (26.24)		66.99 (17.27)	
	Diploma	74.02 (13.50)		65.64 (16.86)		58.67 (19.53)		76.53 (11.85)	
	Academic degree	63.88 (19.67)		59.45 (20.57)		55.28 (22.09)		71.01 (15.21)	
Occupation**	Housewife	72.54 (15.66)	0.021	66.24 (17.12)	0.017	59.76 (18.88)	0.019	76.59 (12.25)	0.007
	Employed	64.59 (19.47)		56.54 (19.34)		49.22 (21.51)		68.70 (15.20)	
	Retired	69.76 (16.71)		66.47 (19.34)		60.44 (20.75)		76.07 (13.34)	
	Self-employed	66.89 (22.66)		60.57 (20.76)		56.25 (23.12)		72.44 (16.83)	
	labor	75.27 (15.45)		63.39 (24.20)		64.43 (19.62)		77.87 (15.32)	
Inhabitant**	Rural	73.71 (13.66)	0.034	67.31 (18.81)	0.492	63.39 (17.39)	0.003	78.24 (12.06)	0.025
	Urban	7.028 (17.84)		64.80 (18.95)		58.59 (20.30)		75.23 (13.76)	
	Outskirts	62.24 (21.41)		64.54 (20.03)		44.59 (33.68)		68.98 (18.75)	
Income**	Good	69.89 (9.63)	0.262	66.80 (18.77)	0.246	66.07 (17.49)	0.127	77.77 (10.92)	0.145
	Medium	70.74 (16.92)		65.20 (17.69)		59.03 (19.76)		75.59 (13.23)	
	Weak	67.50 (18.17)		61.78 (20.11)		55.57 (21.03)		72.76 (14.14)	
The age onset of hypertension **	<30	61.36 (23.19)	< 0.001	56.29 (20.60)	0.001	50.78 (23.88)	< 0.001	68.16 (16.98)	<0.001
	30-60	70.46 (17.59)		64.61 (18.49)		57.60 (20.51)		74.95 (13.65)	
	>60	75.70 (11.96)		69.10 (16.10)		66.07 (15.32)		80.04 (9.92)	
Duration period of Hypertension**	≤ 5	72.17 (15.40)	0.073	63.93 (17.64)	0.055	60.55 (17.65)	0.271	76.06 (11.76)	0.100
	6-10	67.82 (19.93)		62.52 (19.85)		56.60 (22.85)		73.34 (15.65)	
	>10	72.00 (16.85)		67.32 (17.99)		59.44 (20.17)		76.65 (13.29)	
BMI**	<18.5	79.68 (11.03)	0.072	73.01 (14.09)	0.016	65.51 (11.30)	0.519	82.100 (9.65)	0.066
	18.5-24.99	65.88 (19.39)		59.62 (22.02)		56.23 (22.92)		71.88 (15.69)	
	25-29.99	70.87 (17.10)		63.96 (18.42)		59.72 (20.60)		75.47 (13.54)	
	≥30	70.52 (15.57)		67.71 (16.82)		59.45 (20.23)		76.35 (12.47)	

* Independent Samples T-test, ** One-way ANOVA Test.

**Fig 3:**
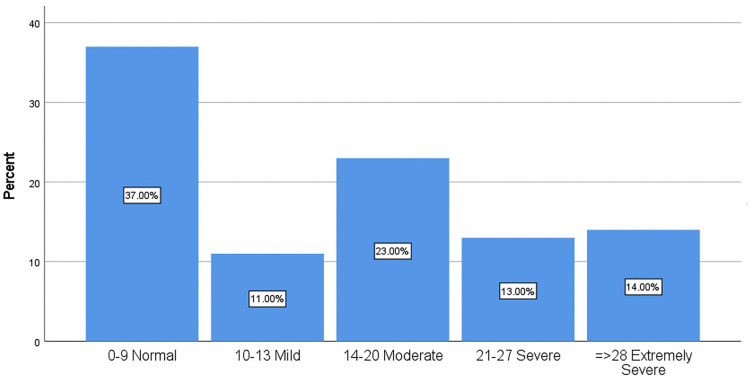
Approximate depression frequencies.

**Fig 4:**
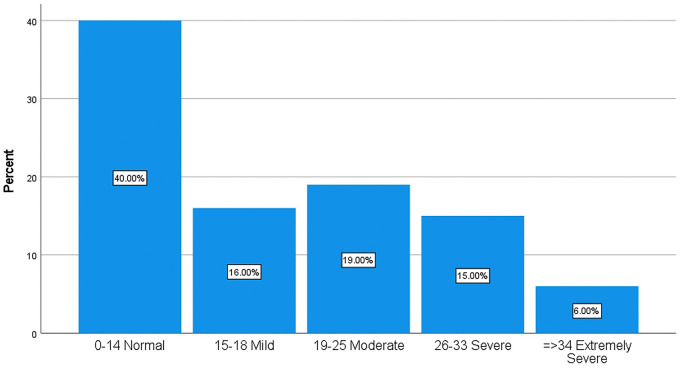
Approximate anxiety frequencies.

**Fig 5:**
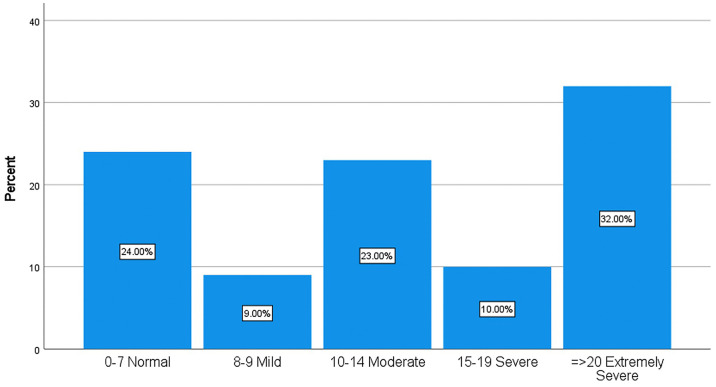
Approximate stress frequencies.

Table 4 showed the frequency of psychological disorders and based on the results 62.5% (n = 318) had depression and only 14.3% (n = 73) had extremely severe depression. Among participants, 75.2% (n = 383) had the anxiety and only 32.2% had extremely severe anxiety. Also, 59.3% (n = 302) had the stress and only 6.7% (n = 34) had extremely severe stress. Other information was mentioned in the Table 4. Also the psychological disorders Frequencies were presented separately in Fig 3, Fig 4, and Fig 5.

**Table 4:** Psychological disorders frequency.

Variables		Depression (n)					Anxiety (n)					Stress (n)				
		Normal	Mild	Moderate	Severe	Extremely Severe	Normal	Mild	Moderate	Severe	Extremely Severe	Normal	Mild	Moderate	Severe	Extremely Severe
Age Group	x < 30	1	0	1	2	1	0	0	1	1	3	0	1	1	2	1
	30-60	82	25	54	30	35	60	14	58	25	69	91	37	49	35	14
	>60	103	35	60	32	36	62	31	57	27	79	111	47	49	41	18
Sex	Men	50	21	36	25	23	40	13	34	15	53	60	28	33	24	10
	Women	138	38	80	42	50	85	33	80	39	111	144	56	67	57	24
Marital Status	Married	155	47	97	48	43	104	34	99	44	109	169	66	75	65	15
	Single	1	0	1	1	4	0	0	1	0	6	0	0	3	1	3
	Divorced	0	0	2	4	3	0	0	1	0	8	0	2	3	3	1
	Widowhood	31	12	16	13	21	20	11	15	8	39	35	15	19	10	14
Income	Good	1	1	9	1	1	3	2	3	2	3	3	4	4	1	1
	Medium	157	46	76	44	31	101	29	88	36	100	164	59	65	54	12
	Weak	14	9	23	14	34	10	9	15	10	50	21	13	24	17	19
Inhabitant	Rural	45	17	20	8	13	22	10	27	16	28	55	12	17	12	7
	Urban	127	40	86	48	48	91	32	82	35	109	132	67	70	58	22
	Outskirts	1	0	5	4	4	1	1	1	1	10	2	2	6	2	2
Education	Illiterate	26	14	21	13	12	14	9	25	9	29	34	19	18	7	8
	Elementary	70	18	33	17	20	42	16	31	16	53	75	17	27	27	12
	Middle school	23	2	14	8	9	14	4	14	4	20	17	16	13	7	3
	High school	4	2	4	3	2	3	2	2	0	8	4	3	3	5	0
	Diploma	27	8	19	7	10	22	6	22	6	15	29	7	11	19	5
	Academic degree	33	15	23	19	16	25	8	19	18	36	39	21	26	15	5
Occupation	Housewife	115	32	66	33	40	69	25	69	31	92	123	44	55	45	19
	Employed	12	7	8	5	5	9	2	6	6	14	13	9	7	6	2
	Retired	40	13	31	20	17	32	12	28	12	37	46	21	28	17	9
	Self-employed	12	4	7	7	7	11	2	8	4	12	12	8	6	8	3
	labor	8	2	3	2	1	3	3	5	0	5	10	1	2	3	0
HTN Duration	≤ 5	64	17	29	19	23	54	11	33	16	38	67	21	30	24	10
	6-10	45	16	23	16	14	30	11	29	9	35	48	18	25	16	7
	>10	71	24	53	24	32	38	22	46	23	75	80	39	39	31	15
All participants		191 (37.5)	60 (11.8)	118 (23.2)	67 (13.2)	73 (14.3)	126 (24.8)	46 (9)	119 (23.4)	54 (10.6)	164 (32.2)	207 (40.7)	86 (16.9)	101 (19.8)	81 (15.9)	34 (6.7)

Table 5 showed Pearson correlation between variables. Based on the results, depression had a positive and significant bidirectional correlation with anxiety (r = 0.797), stress (r = 0.836), and HTN duration (r = 0.108). Also, depression had a negative and significant bidirectional correlation with self-care (r = −0.314) and subscales of maintenance (r = −0.289), monitoring (r = −0.247), management (r = −0.273), and age of onset of HTN (r = −0.098). Anxiety had a positive and significant bidirectional correlation with stress (r = 0.767) and HTN duration (r = 0.190). Also, anxiety had a negative and significant bidirectional correlation with self-care (r = −0.330) and subscales of maintenance (r = −0.323), monitoring (r = −0.209), management (r = −0.319), and age of onset of HTN (r = −0.099) (Table 5).

**Table 5:** Pearson correlation between variables.

Correlations											
		Depression	Anxiety	Stress	Maintenance	Monitoring	Management	Total Self-care	duration	BMI	Age of Onset of HTN
Depression	Pearson Correlation	1	.797^**^	.836^**^	−.289^**^	−.247^**^	−.273^**^	−.314^**^	.108^*^	−.059	−.098^*^
	Sig. (2-tailed)		.000	.000	.000	.000	.000	.000	.019	.239	.034
	N	509	509	509	509	509	509	509	470	397	467
Anxiety	Pearson Correlation	.797^**^	1	.767^**^	−.323^**^	−.209^**^	−.319^**^	−.330^**^	.190^**^	−.026	−.099^*^
	Sig. (2-tailed)	.000		.000	.000	.000	.000	.000	.000	.603	.032
	N	509	509	509	509	509	509	509	470	397	467
Stress	Pearson Correlation	.836^**^	.767^**^	1	−.301^**^	−.217^**^	−.268^**^	−.304^**^	.086	−.013	−.106^*^
	Sig. (2-tailed)	.000	.000		.000	.000	.000	.000	.061	.800	.022
	N	509	509	509	509	509	509	509	470	397	467
Maintenance	Pearson Correlation	−.289^**^	−.323^**^	−.301^**^	1	.634^**^	.604^**^	.857^**^	−.060	.009	.238^**^
	Sig. (2-tailed)	.000	.000	.000		.000	.000	.000	.194	.856	.000
	N	509	509	509	509	509	509	509	470	397	467
Monitoring	Pearson Correlation	−.247^**^	−.209^**^	−.217^**^	.634^**^	1	.581^**^	.858^**^	.019	.104^*^	.183^**^
	Sig. (2-tailed)	.000	.000	.000	.000		.000	.000	.677	.039	.000
	N	509	509	509	509	509	509	509	470	397	467
Management	Pearson Correlation	−.273^**^	−.319^**^	−.268^**^	.604^**^	.581^**^	1	.861^**^	−.085	−.010	.238^**^
	Sig. (2-tailed)	.000	.000	.000	.000	.000		.000	.067	.835	.000
	N	509	509	509	509	509	509	509	470	397	467
Total Self-care	Pearson Correlation	−.314^**^	−.330^**^	−.304^**^	.857^**^	.858^**^	.861^**^	1	−.050	.039	.254^**^
	Sig. (2-tailed)	.000	.000	.000	.000	.000	.000		.284	.443	.000
	N	509	509	509	509	509	509	509	470	397	467
HTN Duration	Pearson Correlation	.108^*^	.190^**^	.086	−.060	.019	−.085	−.050	1	.051	−.270^**^
	Sig. (2-tailed)	.019	.000	.061	.194	.677	.067	.284		.328	.000
	N	470	470	470	470	470	470	470	470	368	466
BMI	Pearson Correlation	−.059	−.026	−.013	.009	.104^*^	−.010	.039	.051	1	−.170^**^
	Sig. (2-tailed)	.239	.603	.800	.856	.039	.835	.443	.328		.001
	N	397	397	397	397	397	397	397	368	397	368
Age of Onset of HTN	Pearson Correlation	−.098^*^	−.099^*^	−.106^*^	.238^**^	.183^**^	.238^**^	.254^**^	−.270^**^	−.170^**^	1
	Sig. (2-tailed)	.034	.032	.022	.000	.000	.000	.000	.000	.001	
	N	467	467	467	467	467	467	467	466	368	467

** Correlation is significant at the 0.01 level (2-tailed).

* Correlation is significant at the 0.05 level (2-tailed).

Table 6 presented the Linear regression in prediction the Hypertension Self-care. According to the table the variables of Depression, Anxiety, Stress, Age, Education and Income could predict 15% of self-care of hypertension variance (Table 6).

**Table 6:** Linear regression in prediction the self-care of hypertension.

Variables	Unstandardized Coefficients		Standardized Coefficients	t	P-value	F	Adjusted R2	P-value
	B	Std. Error	Beta					
(Constant)	76.509	5.253		14.564	.000	14.481	0.156	<0.001
Depression	−.186	.234	−.072	−.798	.425			
Anxiety	−.631	.220	−.222	−2.864	.004			
Stress	−.169	.224	−.063	−.757	.449			
Age	.163	.054	.160	3.038	.003			
Education	−.749	.386	−.104	−1.940	.053			
Income	−.518	1.405	−.017	−.369	.713			

a. Dependent Variable: Self-care of Hypertension.

## Discussion

This study investigated the prevalence of anxiety, stress and depression, and their association with self-care levels in individuals with hypertension. The results generally indicate a substantial burden of psychological distress, notably stress, anxiety and depression among hypertensive patients. Specifically, 62.5% of patients had depression, with 14.3% experiencing severe depression; 75.2% had anxiety, with 32.2% experiencing severe anxiety; and 59.3% had stress, with 6.7% experiencing severe stress. Similarly, Mamoona Mushtaq et al. demonstrated that mental health issues such as depression, anxiety, and stress are frequently observed as comorbidities in hypertensive individuals and contribute to worsened outcomes and quality of life [27]. This finding underscores the importance of screening for and addressing psychological disorders in hypertensive populations as part of clinical care. The psychological disorders prevalence especially anxiety was exceptionally high in this study. The possible reasons for this issue could be the vigilance” effect (anxiety specifically related to medical monitoring or the special Persian translation of DASS-21that the 4-point Likert Persian translation was relatively different by original version.

Beyond the demographic correlates identified in our sample, the present findings may also reflect broader psychological mechanisms described in the international literature. Recent evidence suggests that personality-related factors such as vigilance and Type D traits are associated with greater anxiety and somatization in hypertensive patients, supporting the idea that an enduring tendency toward threat monitoring and negative affectivity may increase psychological distress and reduce effective self-care. In addition, poor emotional regulation, particularly anger suppression and avoidant coping, has been linked to higher levels of anxiety and depressive symptoms, indicating that maladaptive coping styles may further compromise disease management. Finally, the decline in vitality observed in hypertensive populations has been shown to relate to mental distress partly through impaired social functioning, underscoring the protective role of social support and social participation in buffering distress and promoting self-care behaviors. Together, these findings provide a broader framework for interpreting the significant associations observed in our study between marital status, social isolation, psychological distress, and low self-care [29].

A significant negative correlation was observed between the age at which hypertension developed and the severity of anxiety and stress symptoms. The association implies that the developmental timing of hypertension may influence mental health, with younger age at onset correlating with a heavier burden of stress and anxiety. Younger hypertensive patients may face more challenges related to the chronic nature of the disease and its implications for life expectancy and quality of life. The stress of managing a long-term condition, coupled with concerns about potential complications, can contribute to increased anxiety [30]. A meta-analysis by Yu Pan et al. on the association between hypertension and anxiety also indicated that individuals diagnosed at a younger age experience higher psychological pressure [31]. Another study by Nicola Mucci et al., investigating the impact of anxiety and stress on blood pressure, found that younger adults with hypertension often experience greater psychological stress and anxiety compared to older adults [32]. This finding highlights the necessity of addressing mental health challenges in younger hypertensive patients as part of comprehensive disease management.

Regarding the age of hypertension onset and self-care, our study found a significant positive correlation. This implies that a later onset of hypertension is associated with more appropriate self-care practices. Older individuals may have had more time to acquire knowledge, establish consistent routines, and integrate self-management practices—such as medication adherence, dietary control, physical activity, and regular blood pressure monitoring—into their daily lives. Sari et al. reported that older hypertensive patients demonstrate better self-care behaviors, potentially due to greater awareness and longer experience in managing their condition [33]. Kazemi Shishavan et al. also found that self-care adherence tends to be higher among older patients [34].

The analysis revealed a significant positive association between marital status and self-care, indicating that married individuals with hypertension tended to maintain better self-care practices. This aligns with existing literature emphasizing the vital role of social support, particularly spousal support, in boosting adherence to self-care behaviors. Married individuals may benefit from the emotional and practical assistance provided by their partners, such as reminders for medication, encouragement to maintain lifestyle modifications, and shared responsibility in health management, all of which contribute to better hypertension control. Ranak B Trivedi et al. also demonstrated that marital status, as an indicator of social network, is associated with improved hypertension control, primarily through better medication adherence and lower smoking rates [35]. The presence of a supportive spouse can enhance patients’ self-efficacy and motivation, which are crucial for consistent self-care [36]. Furthermore, Azmiardi et al., showed a significant positive correlation between self-care behaviors and family support and in hypertension management [37].

Based on the results, depression had a significant positive correlation with anxiety, stress, and the duration of hypertension. This means that individuals with a longer history of hypertension exhibited higher levels of depression, stress, and anxiety. This finding suggests that the persistent burden of managing a chronic condition like hypertension can increase psychological distress and further exacerbate mental health outcomes. Mushtaq and Najma, in their study on demographic factors affecting blood pressure, also found significant positive relationships between depression, anxiety, stress, and hypertension [27]. Another longitudinal study by Geetha Kandasamy et al. confirmed that long-term hypertension is significantly associated with anxiety and depression [38].

Furthermore, the analysis showed a significant negative correlation between self-care adherence and the severity of both depressive and anxiety symptoms. In other words, individuals with higher levels of depression and anxiety had lower levels of self-care. Psychological distress, particularly in the form of depressive and anxiety symptoms, often impairs an individual’s motivation and capacity to participate in effective self-care activities. Studies across various patient populations consistently indicate that depression and anxiety disrupt self-care routines, likely due to reduced energy, concentration, and self-efficacy necessary for maintaining health practices [39–41].

Finally, the results of linear regression indicated that 15% of self-care variance was predicted by depression, anxiety, stress, age, education and income. Two variables of anxiety and age could significantly predict self-care of hypertension. Anxiety not only could interfere with normal self-care routines, also it could exacerbate hypertension on its own, therefore anxiety screening then providing mental health services by psychologists among hypertensive people seems necessary.

The substantial sample size employed in this study enhances the statistical power and generalizability of our findings. Additionally, the inclusion of diverse predictive factors, encompassing psychological, social, and demographic variables, increased the comprehensiveness and accuracy of the predictions. The dependence on self-report measures constitutes a study limitation, as responses may have been influenced by factors such as social desirability bias or memory inaccuracies.

## Conclusion

This study revealed a high prevalence of anxiety, depression, and stress among patients with hypertension and found significant associations between these psychological disorders and the patients’ self-care levels. The findings indicate that hypertensive individuals with higher levels of depression and anxiety exhibit lower self-care, which can undermine disease management and clinical prognosis. Our results indicate that an earlier diagnosis of hypertension correlates with a heavier psychological burden, characterized by elevated levels of anxiety and stress. These findings call for targeted mental health screening and interventions for this vulnerable patient subgroup. Conversely, older hypertensive patients demonstrated better self-care for various reasons, and married individuals showed higher levels of self-care compared to singles, underscoring the key role of social support. Consequently, a multifaceted strategy incorporating psychological assessment, therapeutic support, and robust social backing is crucial to mitigate the multifaceted burden of hypertension and promote better life quality among affected individuals. In summary, the comprehensive management of chronic diseases should integrally address mental health and social factors to achieve optimal treatment outcomes.

### Clinical implications

From a clinical perspective, the findings of this study suggest that routine screening for psychological distress should be integrated into hypertension care. In addition, combined interventions such as cognitive-behavioral counseling and psychoeducation may be beneficial for improving patients’ psychological well-being and self-management behaviors. Previous evidence indicates that such integrated programs can also contribute to improvements in physical indicators, including BMI, highlighting their potential value in a multidisciplinary approach to hypertension management.

## Abbreviations

DASS-21: Depression, Anxiety, and Stress Scale-21
SC-HI V3: Self-Care of Hypertension Inventory version 3
